# Bilateral male breast cancer with male potential hypogonadism

**DOI:** 10.1186/1477-7819-5-60

**Published:** 2007-06-02

**Authors:** Yukiko Hirose, Mitsunori Sasa, Yoshimi Bando, Toshiyuki Hirose, Tadaoki Morimoto, Yasushi Kurokawa, Taeko Nagao, Akira Tangoku

**Affiliations:** 1 Department of Oncological and Regenerative Surgery, Institute of Health Biosciences, The University of Tokushima, 3-18-15, Kuramoto-Cho, Tokushima 770-8509, Japan; 2 Department of Surgery, Tokushima Breast Care Clinic, 4-7-7, Nakashimada-Cho, Tokushima 770-0052, Japan; 3 Department of Molecular and Environmental Pathology, Institute of Health Biosciences, The University of Tokushima Graduate School, 3-18-15, Kuramoto-Cho, Tokushima 770-8509, Japan; 4 Department of Surgery, National Higashi Tokushima Hospital, 1-1, Ohmukai-kita, Ootera, Itano, Tokushima 779-0193, Japan; 5 School of Health Sciences, University of Tokushima, 3-18-15, Kuramoto-Cho, Tokushima 770-8509, Japan; 6 Department of Urology, Tsurugi Municipal Handa Hospital 234-1, Nakayabu, Handa, Tsurugi-cho, Tokushima 779-4401, Japan

## Abstract

**Background:**

Male breast cancer is a comparatively rare disease, and simultaneous bilateral male breast cancer is considered to be an extremely rare event. Risk factors are said to be genetic factors and hormonal abnormalities due to obesity or testicular diseases.

**Case presentation:**

The patient was a 47-year-old Japanese male. His family had no history of female breast cancer. This patient also had hypospadias and hormonal examination indicated the presence of primary testicular potential hypogonadism, and these hormonal abnormalities seemed to be present since childhood or the fetal period. The bilateral breast cancer developed in this man at a comparatively young age, and histopathological studies of multiple sections showed that there was almost no normal epithelial cell in the ducts, while the ducts were almost completely filled with breast cancer cells.

**Conclusion:**

It is thought that male breast cancer is caused by an imbalance between estrogen and testosterone. We cannot rule out the possibility that the breast cancer developed due to the effect of the slight elevation of estrogen over a long period of time, but the actual causative factors in this patient were unable to be definitively identified. In the future, we hope to further elucidate the causes of male breast cancer.

## Background

Male breast cancer (MBC) is a comparatively rare disease, accounting for 1% or less of all male cancers and only about 1% of all breast cancers [1-4]. In addition, bilateral breast cancer accounts for only 1.5–2% of MBC, and simultaneous cancers is extremely rare [5-8]. The consensus is that MBC is caused by an imbalance between estrogen and testosterone [4,9]. Various risk factors have been proposed for MBC, including BRCA2 gene anomalies [10], Klinefelter's syndrome [11], a genetic background such as a familial history of breast cancer, hormonal abnormalities due to obesity or testicular disease (cryptorchidism, mumps, orchitis, orchiectomy), exposure to radiation [1-4,9]. Here, we report a case of simultaneous bilateral breast cancer diagnosed in a Japanese male. This case is discussed in relation to the published literature.

## Case presentation

**Figure 1:**
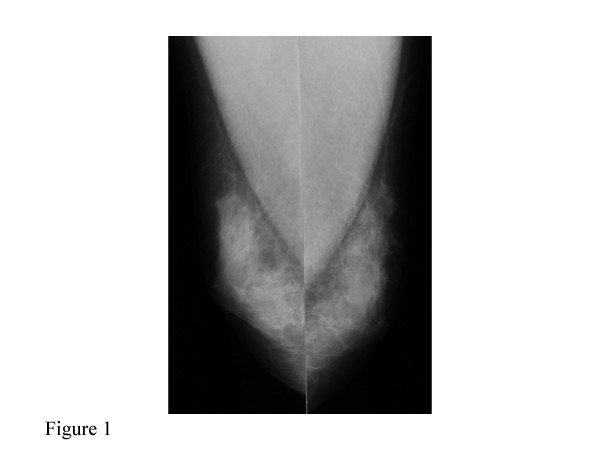
Mammography revealed scattered microcalcifications in the left breast.

**Figure 2:**
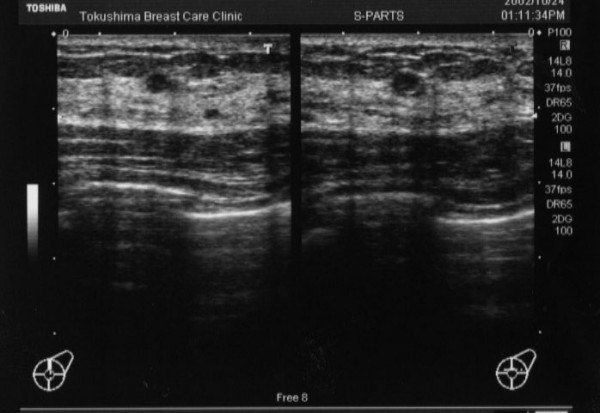
Ultrasonography showed a hypo-echoic legion of 3 mm in diameter in C region of the left breast.

A 47-year-old Japanese male who worked for a food company, presented with complaint of a bloody discharge from the left nipple. This patient also had hypospadias, a condition that also existed in one male relative. There was no familial history of breast cancer. The patient had undergone plastic surgery for the hypospadias two times at the age of 4–5 years. Then, at the age of 20–21 years the patient underwent four more plastic surgery operations for the hypospadias. The patient was male in appearance. He has male sterility and had no history of trauma to the external genitalia or mumps. As the history of the present disease, the patient noticed a bloody discharge from the left nipple and thus came to the authors' hospital for examination. The body height was 175 cm, the body weight was 80 kg, and with a BMI of 26.1 the patient showed a slight tendency to obesity. Examination yielded local findings of a bloody discharge from a single duct of each of the bilateral nipples. Palpation did not detect any clear mass in either of the breasts or their areolas. The axillary lymph nodes were not palpated. Mammography revealed scattered microcalcifications in the left breast (Figure 1). There were no abnormal findings for the right breast. Ultrasonography showed a hypo-echoic lesion of 3 mm in diameter in C region of the left breast (Figure 2), and the right breast showed duct dilatation in C region. Fine-needle aspiration cytology showed class V for both the left and right breasts, and smear cytology also showed class V for both breasts.

**Figure 3:**
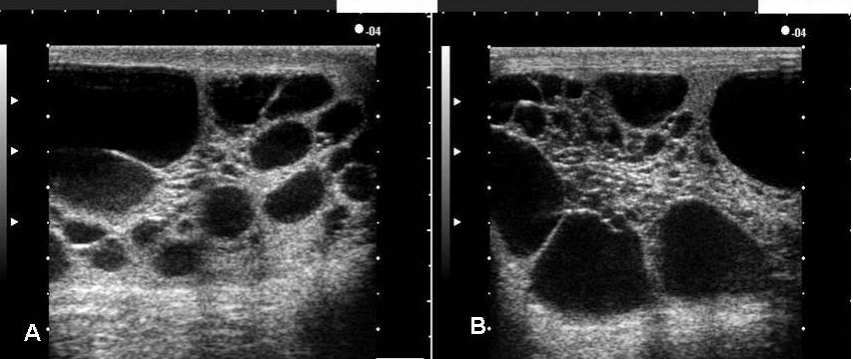
Ultrasonography showed that both testes had undergone overall degeneration to multilocular cysts, and there was overall enlargement of the testes.

Blood tests showed no abnormalities. Hormone tests gave the following results: LH 25.5 mIU/ml (normal range: 1.1~8.8), FSH 33.6 mIU/ml (1.8~13.6), PRL 18.8 ng/ml (3.6~12.8), E1 96.2 pg/ml (10~90), E2 28.1 pg/ml (20~60.1), E3 ≤ 5 pg/ml (≤ 5), testosterone 4.11 ng/ml (2.7–11) and free-testosterone 9.1 pg/ml (6.9–18.4). Thus, LH and FSH were high, and PRL was slightly elevated. On the other hand, E1 was slightly elevated, whereas E2, E3, testosterone and free testosterone were normal. In the LHRH loading test, LH and FSH increased normally, but testosterone and estrogen showed poor responses. In the hCG loading test (briefly, the measurement of testosterone was performed about 2 h after loading of hCG 10000 IU/body), testosterone showed a baseline (preloading) value of 4.82 ng/ml and then increased only slightly to 7.41 ng/ml after loading. This result indicated the presence of primary potential hypogonadism. In addition, a chromosomal study showed a normal male karyotype of 46XY. Considering the history of hypospadias, the patient was referred to the Urology Department for detailed tests relating to male sexual function insufficiency, sexual differentiation abnormality. The Urology Department found the patient to have a normal male form in terms of the outward appearance, head of hair, hair growth in the inguinal region, but at the penile-scrotal angle there was scarring from the surgery for the hypospadias. Palpation revealed an elastic, soft, testicle-like portion in the bilateral scrota. Ultrasonography showed that both testes had undergone overall degeneration to multilocular cysts, and there was overall enlargement of the testes (Figure 3). Pelvic MRI examination showed the bilateral scrota to be filled with a water-density mass, and there were no clear findings characteristic of testes.

On the basis of the above findings, a diagnosis of male bilateral breast cancer accompanied by male potential hypogonadism was made. Simple mastectomy was performed on both breasts.

**Figure 4:**
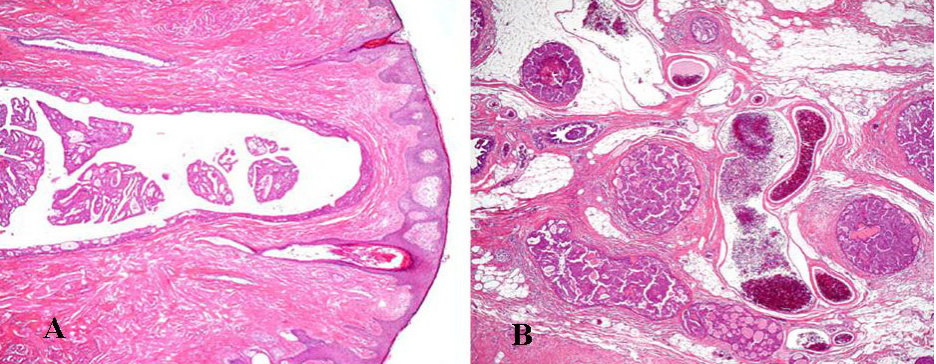
Carcinoma cells in a large lactiferous duct of the subareolar region (A)(×20). Noninvasive ductal carcinoma forming cribriform or micropapillary patterns, but some ducts containing comedonecrosis (B)(×20).

**Figure 5:**
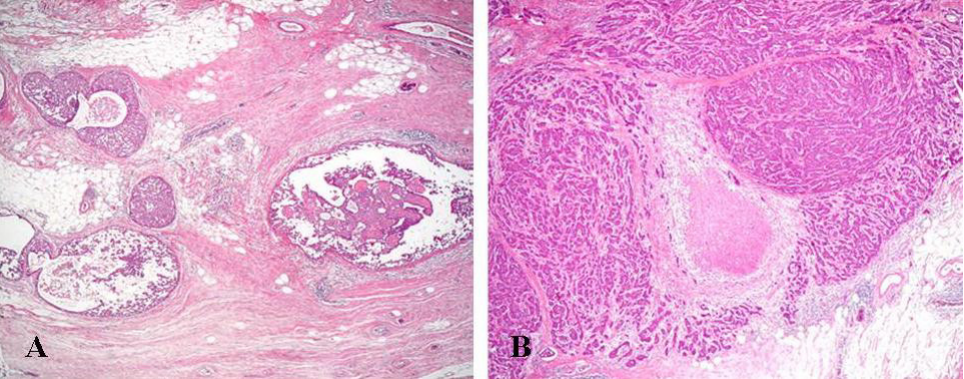
Noninvasive ductal carcinoma, micropapillary and cribriform(A)(×20). Invasive ductal carcinoma with small coagulative necrosis (B)(×20).

Histopathological studies showed proliferation of clearly atypical cells in the duct of the left breast, and that was accompanied by partial necrosis. A diagnosis of noninvasive ductal carcinoma was made (Figure 4). In addition, immunohistochemical staining of the left breast showed the tumor to be estrogen receptor (ER) (+ve), progesterone receptor (PgR) (+ve) and HER2/neu(-ve). In the right breast, most of the atypical cell proliferation was in the duct, but there was also some invasion of the stroma by atypical cells, leading to a diagnosis of invasive ductal carcinoma, solid-tubular type (partial papillotubular type). As in the left breast, there was partial necrosis (Figure 5), and the immunohistochemical staining results were again ER (+ve), PgR (+ve) and HER2/neu (-ve). In addition, inspection of multiple sections from the bilateral breasts found almost no normal epithelial cells in the ducts, and the insides of the ducts were almost totally filled with cancerous issue.

Postoperatively, endocrine therapy consisting of tamoxifen 20 mg per day is being administered, and as of this writing there has been no evidence of recurrence of the malignancy.

## Discussion

MBC is considered to be a rare disease, and bilateral, simultaneous MBC is extremely rare [1-4]. MBC is said to be more common in elderly patients compared with the disease in females [9]. It has been reported that the characteristics of MBC are similar to those of postmenopausal female breast cancer [3], but there are also reports that hormonal receptor studies and studies of the therapeutic efficacy of aromatase inhibitors indicate MBC to differ from female breast cancer [12-15]. Various risk factors have been pointed out for MBC, including BRCA2 gene anomalies [10], Klinefelter's syndrome [11], a genetic background such as a familial history of breast cancer, hormonal abnormalities due to obesity or testicular disease (cryptorchidism, mumps, orchitis, orchiectomy), exposure to radiation [1-4]. With regard to hormonal abnormalities, it has been reported that MBC is caused by an imbalance between estrogen and testosterone [4,9]. Breast cancer has been observed to occur in patients who were administered an estrogen for a long period of time [1], and estrogen is thought to play an important role in the development of MBC since the plasma concentration of this hormone is higher in MBC patients than in normal males [1-3,9]. It is said that the production, metabolism and bioavailability of estrogen are enhanced in the subcutaneous tissues of obese males as a result of activation of aromatase, a decrease in sex-hormone-binding globulin (SHBG) and for these reasons obesity has been reported as a risk factor for MBC [2]. On the other hand, with regard to testosterone, castration for the purpose of gender transformation or prostate gland therapy is said to increase the risk of MBC, while testicular dysfunction due to mumps or cryptorchidism alters the levels of estrogens, androgens and gonadotropins and is also thought to increase the risk of MBC [3]. Prolactin is involved in the differentiation and development of normal breast tissue, and there have been reports of bilateral breast cancer in patients with pituitary tumor, especially when accompanied by hyperprolactinemia [6,7]. Also, because administration of prolactin has been shown to cause breast cancer in animal studies, this hormone is thought to be strongly involved in the development of this malignancy [16]. However, prolactin is produced in an autocrine and/or paracrine manner by breast cancer cells and normal breast cells, and it is said that its activity is expressed only at the cellular level. Thus, it has been judged that it does not exert any influence on prolactin production by the pituitary, and that pituitary prolactin production is not involved in the development of breast cancer [17].

BRCA2 testing was not performed on our present patient. However, since there was no familial history of breast cancer, it can be surmised that the cause of the disease was the hormonal disturbances rather than genetic factors. Moreover, the facts that this patient was rather young (47 years old) to have this disease and that the disease was simultaneously bilateral point to the existence of high risk factors.

A chromosomal study showed the patient to have a normal male karyotype of 46XY, but he had complications of hypospadias and male potential hypogonadism. Hypospadias is one of the most commonly occurring congenital abnormalities, and one of its causes is thought to be hormonal abnormalities during the fetal stage [18]. In addition, although androgen gene and/or androgen receptor gene anomalies might be thought to be involved [3], this patient showed only a mild degree of abnormality of the external sex organs and presented a normal male appearance in terms of the body type, development of body hair. Also, the fact that there was only a slight increase in the testosterone level following a loading test provides further evidence negating involvement of these two genetic anomalies in this patient. However, there is a possibility that the hypospadias was familial in nature, and thus we cannot rule out the possible existence of other genetic anomalies in the patient.

In an attempt to elucidate the causative mechanism of the bilateral breast cancer in this Japanese male, we performed various hormone tests. Imaging studies found almost no normal testicular tissue, but in the hCG loading test testosterone increased even if only slightly, and although the hormone secretion function of the testes was disturbed, the function can nevertheless be considered to have been about half of normal. This suggests that a partial Leydig cell function of cyst wall do exist. It can be surmised that this was because stimulation by LH and FSH due to feedback arising from the testicular potential hypogonadism caused secretion of testosterone from testes and from adrenal glands and thus maintained a normal serum testosterone level.

In addition, since the patient showed a tendency to obesity, the finding that the E1 level was slightly increased even though the testosterone level was normal can be thought to have been a result of estrogen production via aromatase in the adipose cells. We did not measure the level of SHBG, but it can be assumed that SHBG was nearly normal since the level of free testosterone was normal. An SHBG abnormality would affect the estrogen activity, but there was no striking evidence of such an effect in this patient. There is doubt regarding whether a slight elevation of only E1 in men has any clinical significance. Pathologically, the disease developed bilaterally in this patient, and histopathological studies of multiple sections of the resected specimen found almost no normal ductal tissue and almost entirely breast cancer cells. We cannot exclude the possibility that the breast cancer in this patient developed only due to these hormonal abnormalities. Measurements were carried out three times at set intervals, but each time the PRL level was slightly elevated. As noted earlier, PRL has been ruled out as a cause of breast cancer [17]. However, there have been reports of bilateral MBC accompanied by hyperprolactinemia [6,7], and in this light we cannot exclude the possibility that PRL may indeed be somehow involved. In addition, there have been reports that MBC clearly differs from postmenopausal female breast cancer, including recommendation of use of a gonadotropin-releasing hormone analog to interrupt the feedback from the pituitary that occurs at the time of administration of an aromatase inhibitor as therapy for breast cancer in males [12,13]. In the future, we hope to further elucidate the causes of MBC.

## Conclusion

Risk factors for male breast cancer are said to be genetic factors, hormonal abnormalities. The Japanese male patient reported here had developed bilateral breast cancer at a comparatively young age, and in the absence of a familial history of breast cancer it was surmised that the disease had been caused by hormonal abnormalities rather than genetic factors. The patient has male potential hypogonadism that was accompanied by hypospadia. The possibility that the breast cancer developed due to the effect of the slight elevation of estrogen over a long period of time cannot be ruled out, but the actual causative factors in this patient were unable to be definitively identified. In the future, we hope to further elucidate the causes of MBC.

## Competing interests

The author(s) declare that they have no competing interests.

## Authors' contributions

**YH, MS, TH **and **YK **took part in the care of the patient. **YB **examined surgical specimen and took photomicrographs of the slides. **MS **initiated and co-wrote the paper with **YH, TM, TN **and **AT**. All authors read and approved the final manuscript.
